# A single mutation in the PrM gene of Zika virus determines AXL dependency for infection of human neural cells

**DOI:** 10.1128/jvi.01873-24

**Published:** 2025-03-10

**Authors:** Renu Khasa, Sarah C. Ogden, Yuqing Wang, Zongiun Mou, Anna D. Metzler, Xuping Xie, Xinghong Dai, Hengli Tang

**Affiliations:** 1 Department of Biological Science, Florida State University 123386 , Tallahassee, Florida, USA; 2 Department of Physiology and Biophysics, Case Western Reserve University 465580 , Cleveland, Ohio, USA; 3 Department of Microbiology and Immunology, University of Texas Medical Branch at Galveston 547647 , Galveston, Texas, USA; Wake Forest University School of Medicine, Winston-Salem, North Carolina, USA

**Keywords:** virus-host interactions

## Abstract

Zika virus (ZIKV) is spread by mosquito bites and is unique among known flaviviruses for being able to cause microcephaly. Entry factors for ZIKV are incompletely understood, but phosphatidylserine (PS) receptors, including the TAM (Tyro3, AXL, and Mer) and TIM (T-cell Ig mucin) families, can serve as cofactors for flavivirus entry in a cell type-specific manner. We identify AXL as the top hit in a CRISPR/Cas9 genome-wide screen in human glioblastoma cells and establish a definitive role of AXL, but not TYRO3 or MerTK, for ZIKV infection. Additionally, Spondweni virus also shows AXL dependency, while dengue virus infection is not affected by AXL knockout. Passage of ZIKV in AXL knockout (KO) cells generated a mutant virus capable of infection via AXL-independent mechanisms, and multiple independent selections identified a common mutation, H83R, in the prM coding region of the ZIKV genome. The mutant virus exhibits an increased infectivity rate in AXL KO cells as compared to wild-type ZIKV and is dependent upon the single H83R mutation. The mutant virus’ ability to infect cells in an AXL-independent manner is unrelated to interferon signaling antagonism but likely pertains to a change in virus maturation that leads to a structural disturbance of the ZIKV virion. Our study provides evidence for a potential mechanism linking the viral structural proteins and host PS receptor usage during flavivirus infection.

**IMPORTANCE:**

A major challenge in elucidating the mechanism of Zika virus (ZIKV) pathogenesis is the multitude of cell types it infects with distinct requirements. The role of phosphatidylserine (PS) receptors in ZIKV infection is cell type-specific, and the controversy surrounds their function in flavivirus entry. Here, we establish a definitive requirement of AXL for infection of human glioblastoma cells by both Zika and Spondweni virus. We then identified a single amino acid mutation (H83R) in the prM protein of ZIKV that allowed AXL-independent infection of these cells. The H83R-mediated escape of AXL requirement is independent of interferon (IFN) signaling suppression by AXL; instead, the mutation has the potential to disrupt the virus assembly and virion structure. This study reveals a previously unknown connection between the PS receptor usage and the flavivirus prM gene, which can guide detailed molecular mechanism studies of the interplay between virion assembly and virus entry.

## INTRODUCTION

Zika virus (ZIKV) is a mosquito-borne virus and a member of the *Flaviviridae*, which also includes other significant human pathogens, such as West Nile virus (WNV), yellow fever virus (YFV), dengue virus (DENV), and Japanese encephalitis virus (JEV) (1). ZIKV is also closely related to a less studied flavivirus, Spondweni virus (SPONV), the sole other member within the Spondweni serogroup (2). Despite the discovery of the first isolate of ZIKV, MR766, over 50 years ago (3), it was not until after a series of outbreaks in the Pacific islands, culminating in a major outbreak in Brazil in 2015, that ZIKV became a focus of molecular and clinical research. The 2015 outbreak in the Americas revealed a previously undetected pathology of ZIKV, namely the vertical transmission of the virus and the manifestation of congenital birth defects in infants born to infected pregnant women (4). ZIKV infection in adults can also result in neurological diseases, including Guillain-Barre syndrome, acute myelitis, encephalitis, and polyneuropathies, as well as chorioretinitis (4).

ZIKV has a positive-stranded RNA genome of approximately 11 kB with a long open reading frame (ORF) flanked by 5′ and 3′ untranslated regions. The ORF is translated into a single polypeptide which is then cleaved by viral and host proteases into 10 discrete viral proteins. The structural proteins (C, prM/M, and E) participate in the formation of the infectious virion containing the RNA genome, and the nonstructural proteins (NS1, NS2A, NS2B, NS3, NS4A, NS4B, and NS5) are responsible for coordinating RNA replication and antagonizing the cellular antiviral response (5). In addition to the viral proteins, host factors hijacked by the virus play critical roles in the ZIKV life cycle and pathogenesis. The ability to utilize cell type-specific cofactors to enable infection of cells ranging from placental to neuronal lineages potentially contributes to ZIKV’s unique ability among flaviviruses to transmit vertically and cause microcephaly. Prior studies indeed revealed both conserved and cell type-specific proviral factors for ZIKV (6–10). A prominent example of the latter group is AXL, a member of the TAM family of phosphatidylserine (PS) receptors that derive its name from the three constituent members, Tyro3, AXL, and MerTK (11–14). These receptor tyrosine kinases function in conjunction with their respective soluble ligands, Gas6, or Protein S (15–19) to mediate cell signaling and function in diverse cellular processes. From the many studies investigating the potential role of AXL in ZIKV infection, it is clear that the AXL requirement is model dependent, as even studies using definitive ablation methods such as gene knockout produced different results in distinct infection models (6, 9, 20–38). Moreover, among the results indicating an important role for the involvement of PS receptors in flavivirus infection, there is disagreement on the mechanism of action, with hypotheses ranging from PS-mediated attachment/entry (25, 39–41) to the suppression of the host antiviral IFN response (31–33, 42).

In this report, we establish an essential role of AXL during ZIKV infection of human glioblastoma cells, a proposed target cell type for potential oncolytic viral therapy (10, 43–47). Furthermore, we isolated a ZIKV mutant that bypasses the AXL-dependent mechanism for infection of these cells and then mapped the mutation to the pr region of the prM gene. The single prM mutation does not impact AXL-mediated IFN suppression but alters the virion structure.

## RESULTS

### AXL is an important host factor for ZIKV infection in SNB-19 cells

**Fig 1:**
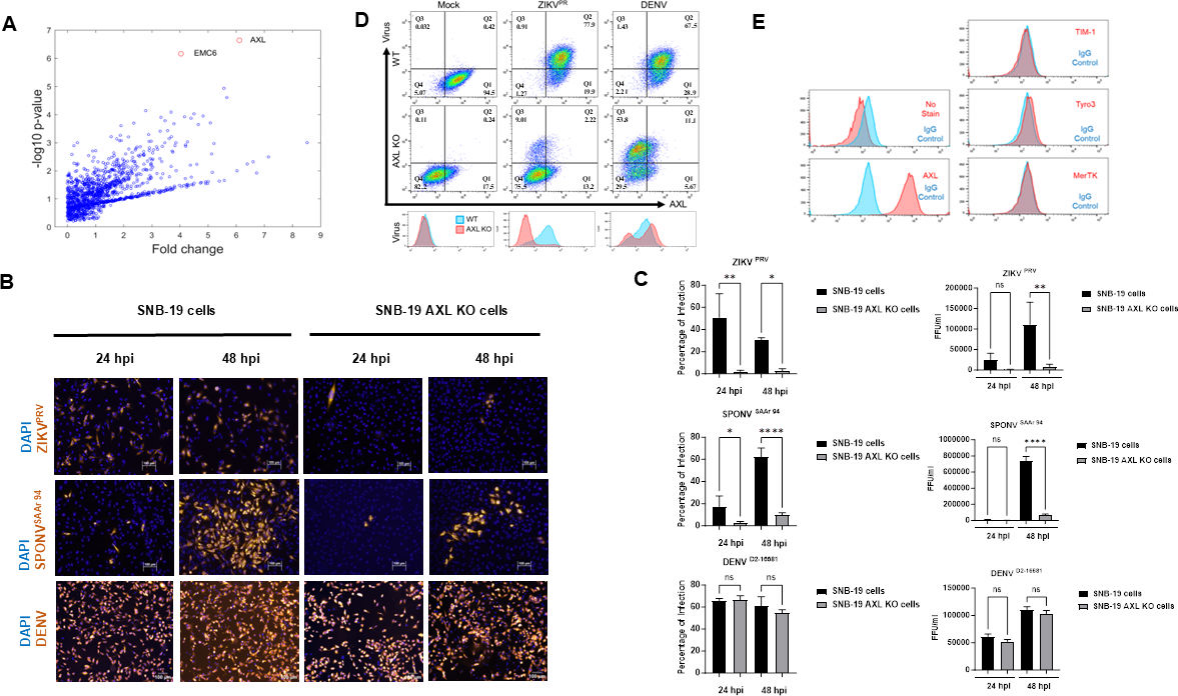
Identification and validation of AXL as a host factor for ZIKV infection in SNB-19 cells. (**A**) Volcano plot of identified gene targets represented as the fold increase of gene target identification relative to uninfected SNB-19 cells as determined by MaGECK analysis. (**B–D**) Knockout of AXL inhibits ZIKV but not DENV infection. AXL KO and WT cells were infected with ZIKV^PRV^, SPONV^SAAr 94^, or DENV2-16681 at a multiplicity of infection (MOI) of 1. Cells were fixed for imaging (**B**), the supernatant was collected for focus-forming units (FFU) at 24 or 48 hpi (**C**), and cells were collected for fluorescence-activated cell sorting (FACS) at 24 hpi (**D**). Quantification of imaging (C left) and FFU (C right) showing a significantly decreased level of ZIKV or SPONV infection in AXL KO SNB-19 cells at 48 hpi as compared to in SNB-19 WT cells but showing no significant change in DENV infection in either cell line (**D**). (**E**) SNB-19 cells do not express other TAM receptors. WT or AXL KO SNB-19 cells were fixed and stained with antibodies for TIM-1, Tyro3, MerTK, AXL, or IgG control prior to analysis by flow cytometry (E, left), and cells were lysed for western blot after 24 hpi (E, right). SNB-19 cells do not express other TAM receptors. One-way analysis of variance (ANOVA) with Tukey’s multiple comparisons test. Three technical replicates. Results are mean ± SEM. *P < 0.05, **P < 0.01, ***P < 0.001, and ****P < 0.0001.

To establish a genome-wide CRISPR/Cas9 screening condition for proviral factors, we sought to identify cell lines from the NCI-60 panel that are readily killed by DENV and ZIKV infection *in vitro*. We selected the SNB-19 glioblastoma line for the screening as infection of these cells with the MR766 (ZIKV^MR^) strain of ZIKV resulted in >99% cell death by 7 days post-infection (data not shown), providing a low background for analysis of critical host factors using a live/dead selection. Following transduction with a lentiviral GeCKO library and ZIKV^MR^ infection, surviving colonies were harvested, and individual single guide RNAs (sgRNAs) were sequenced. Next-generation sequencing was also performed at the steps of library amplification, post-transduction, and post-infection to ensure sgRNA coverage and representation. Model-based Analysis of Genome-wide CRISPR/Cas9 Knockout (MaGECK) was used to evaluate significantly enriched gene targets in surviving cells post-ZIKV infection. AXL was overwhelmingly the most enriched CRISPR target in the surviving cell colonies, followed by EMC6 (Fig. 1A; Table 1), which has been identified in most of the reported screens for flaviviruses (6–8).

**TABLE 1:** Significant and top-rank gene targets from the GeCKO library screen^*a*^

Gene ID	Pos/rank	Pos/score	Pos/*P*-value	Pos/FDR	Num sgRNA	Pos/good sgRNA	Pos/lfc
AXL	1	1.8036E-10	2.2827E-07	0.00495	6	6	6.1136
EMC6	2	9.6399E-08	6.8481E-07	0.007426	6	6	4.0349
EMC1	3	2.1259E-06	0.000011642	0.084158	6	4	5.5622
EMC3	4	4.6911E-06	0.000024881	0.134901	6	5	5.6657
DDHD2	5	0.000013174	0.000072818	0.244637	6	3	2.7856
EMC4	6	0.000018497	0.000094276	0.244637	6	5	4.4981
FFAR3	7	0.000019642	0.000081949	0.244637	6	5	3.6839
DDX3X	8	0.000023921	0.0001203	0.244637	6	4	4.1634
SSR3	9	0.00002494	0.00012532	0.244637	6	5	3.3241
PCDHB15	10	0.000026146	0.00011254	0.244637	5	3	4.8223

^*a*^ Significant gene targets highlighted in green. FDR, false discovery rate; lfc, log fold change.

To validate the GeCKO library sequencing results, we generated SNB-19 cells with AXL KO and subjected them to infection by the ZIKV ^PRV^ (ZIKV PRVABC59) isolate of ZIKV or DENV-2 (D2-16681). While DENV-2 was able to infect both the WT and the AXL KO cells equally (Fig. 1B through D), infection by ZIKV was markedly reduced in the KO cells. Interestingly, infection of AXL KO cells by SPONV was also suppressed (Fig. 1B and C), suggesting that infection of these glioblastoma cells by SPONV also requires AXL.

To determine whether DENV infection of the SNB-19 AXL KO cells was mediated by related PS receptors (39, 48–50), we examined SNB-19 cells for the relative expression level of these proteins. Flow cytometry and western blot analysis revealed that SNB-19 cells do not express the other two TAM members, Tyro3 and Mer, or do they express a similarly implicated PS receptor, TIM-1 (Fig. 1E). Furthermore, AXL KO did not induce any compensatory expression of these PS receptors. These results indicate that, in contrast to ZIKV, DENV infection of the SNB-19 cells likely occurs through a PS-receptor-independent pathway.

### AXL is unique among the TAM family members for mediating ZIKV infection

**Fig 2:**
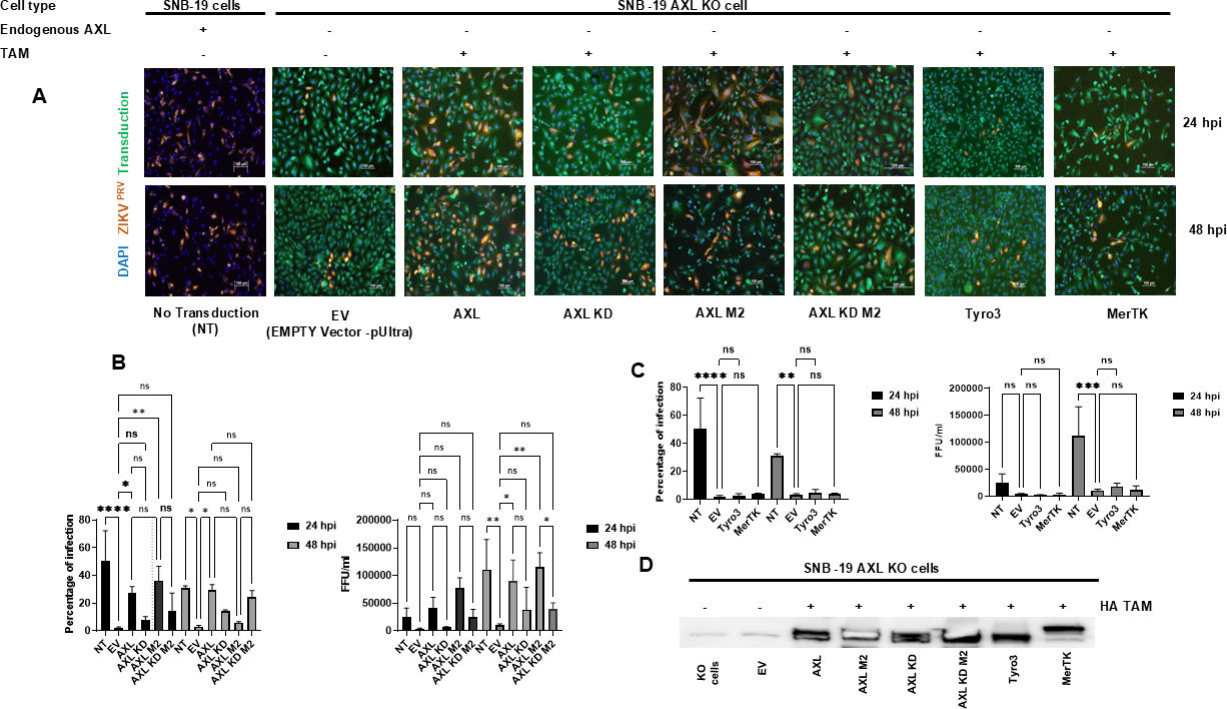
AXL kinase, but not Tyro3 and MerTK, rescues ZIKV infection in AXL KO cells. WT or AXL KO SNB-19 cells were transduced with the indicated lentivirus, followed by ZIKV infection at MOI 1. Cells were fixed for imaging (same images of SNB-19 WT infected with ZIKV were used in Fig. 1 and 2 to show the comparison between transduced and untransduced cells in Fig. 2) (**A**) and quantification (B and C, left). The supernatant was collected for focus-forming units (FFU) after 24 or 48 hpi for the determination of viral titers (B and C, right). (**D**) Expression of the TAM receptors in the KO cells after lentiviral transduction. An anti-HA antibody was used to detect the HA-tagged TAM receptors in the AXL KO SNB-19 cell. For sections B and C, one-way ANOVA with Tukey’s multiple comparisons test was performed. Three technical replicates. Results are mean ± SEM. *P < 0.05, **P < 0.01, ***P < 0.001, and ****P < 0.0001.

We next determined if expressing the various PS receptors in SNB-19 AXL KO cells could restore ZIKV infection. Lentiviral vectors expressing WT AXL, a kinase-dead mutant of AXL (*AXL N90G* and *AXL-KD*), Tyro, and Mer were delivered into the AXL KO cells individually, followed by ZIKV infection. The AXL cDNAs were delivered in both the unmodified form and a modified version (AXL M2) that contained synonymous mutations within the sgRNA recognition site to avoid the potential effect of the stable CRISPR/Cas construct initially delivered to the KO cells. As shown in Fig. 2, re-expressing AXL, but not the kinase-dead mutant, was able to significantly rescue ZIKV infection. These results demonstrate an important role of AXL and its kinase activity in facilitating ZIKV infection. In contrast, expression of Tyro or Mer tyrosine kinases did not rescue infection despite similar expression levels (Fig. 2D), suggesting that AXL is the only TAM family member that can function to mediate ZIKV infection of these glioblastoma cells.

### AXL dependency tracks with structural proteins

**Fig 3:**
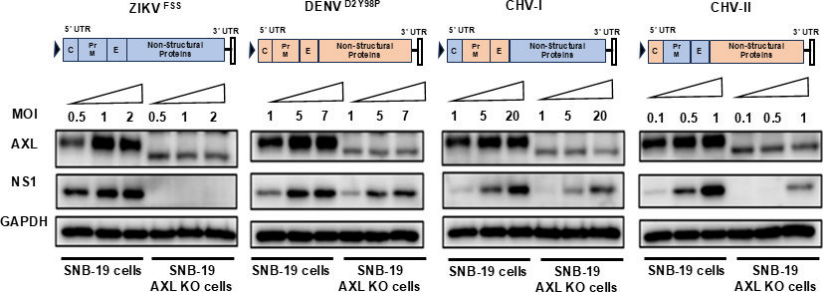
AXL dependence tracks with the structural genes of ZIKV. Schematic of the chimeric viruses are shown at the top of the data panels. SNB-19 cells, WT or AXL KO, were infected with ZIKV, DENV, and chimeric viruses at increasing MOI, and the cell lysates were collected 24 hpi for western blot analysis. The MOIs were kept the same for each virus infecting either the WT or the AXL KO cells, although the MOI needed for each virus to produce a robust NS1 signal on the western blots varied. The AXL KO lysates always showed a few background bands that do not correspond to the correct size of AXL as shown for the WT cells.

To determine if the difference in AXL dependency between ZIKV and DENV infection is mediated by virus-intrinsic differences, we utilized a ZIKV/DENV chimeric virus panel derived from cDNA clones of ZIKV FSS13025 (ZIKV^FSS^) and DENV-2 Y98P (D2-Y98P) (51–54). These chimeric viruses have combinations of the structural proteins of each respective virus, with the first containing the DENV prM/E proteins in the ZIKV backbone (chimeric virus I, CHV-I) and the second containing the inverse arrangement of viral proteins (CHV-II; Fig. 3). Although the infectivity of these chimeric viruses varied greatly and resulted in different ranges of MOI used to achieve infection, the relative difference in AXL dependency was recapitulated with these additional strains of ZIKV and DENV. D2-Y98P infected AXL KO and WT SNB-19 cells with near equal efficiency, while ZIKV^FSS^ infection of AXL KO cells was dramatically reduced as compared to that of WT cells (Fig. 3). Importantly, CHV-I infection of AXL KO cells resulted in only a minimal reduction in viral protein as compared to WT cells, while CHV-II only showed infection in AXL KO cells at higher MOIs and more closely tracked with the infection phenotype of ZIKV. These results indicate that the viral determinants of AXL dependency likely reside within the structural proteins of these flaviviruses. It is also worth noting that both CHV-I and D2Y98P required very high MOI to fully infect WT SNB-19 cells, while ZIKV and CHV-II required much lower MOI for similar infection levels, further suggesting that the infection efficiency of these glioblastoma cells tracks with the respective prM/E proteins.

### Selection and characterization of an AXL-independent ZIKV mutant virus

**Fig 4:**
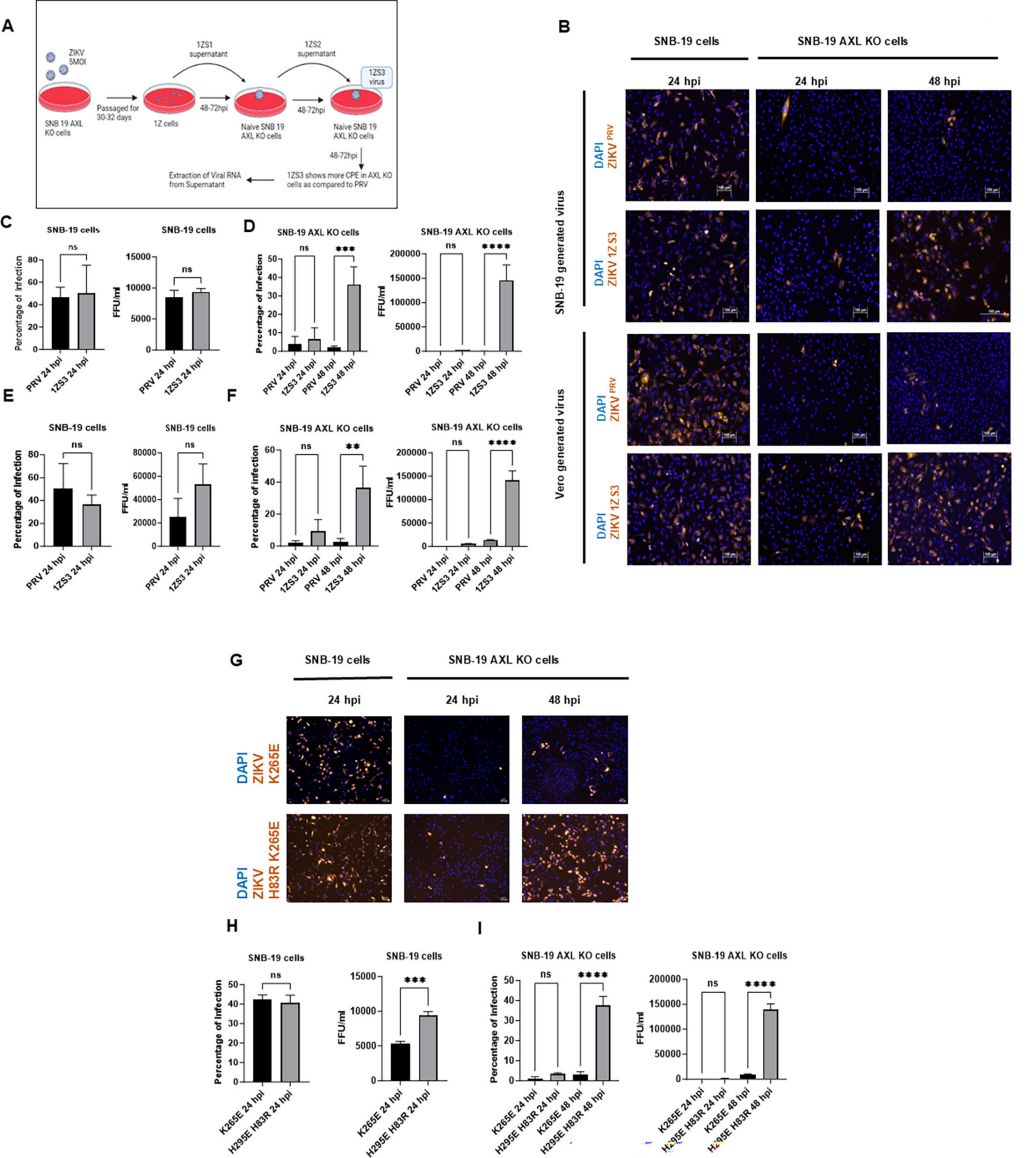
Selection of a ZIKV mutant with reduced dependence on AXL for infection. (**A**) Schematic of the selection. AXL KO SNB-19 cells were infected with ZIKV at MOI 5 and then passaged for 30–32 days, resulting in the generation of 1Z cells, capable of producing ZIKV. The supernatant collected from these cells was used to infect naïve KO cells for three more rounds, and the virus collected at the end is designated 1ZS3. (**B–F**) Differential infection of the AXL KO cells by the 1ZS3 virus. WT or AXL KO SNB-19 cells were infected with ZIKV ^PRV^ or 1ZS3 produced from AXL KO SNB-19 cells (same images of SNB-19 WT and AXL KO SNB-19 cells infected with ZIKV^PRV^ were used in Fig. 1 and 4 to show the comparison between WT and mutant virus infection; B-top; **C and D**) or Vero cells (B-bottom, E, and F) at MOI 1. Cells were fixed for imaging (**B**), and supernatant was collected for focus-forming units (FFU) at 24 hpi in SNB-19 cells and 24 or 48 hpi in AXL KO cells. Quantification of imaging (left of C, D, E, and F) and FFU (right of C, **D, E, and **F) in SNB-19 (**C and D**) or AXL KO cells (**E and F**). (**G–I**) WT or AXL KO SNB-19 cells were infected with K265E or K265E H83R virus at MOI 1. Cells were fixed for imaging (**G**), and supernatant was collected for FFU after 24 hpi in SNB-19 cells and 24 or 48 hpi in AXL KO cells. Quantification of imaging (left H and I) and FFU (right H and I) in SNB-19 cells (**H**) or AXL KO SNB-19 cells (**I**). For sections C, D, E, F, H, and I, one-way ANOVA with Tukey’s multiple comparisons test was performed. Three technical replicates. Results are mean ± SEM. *P < 0.05, **P < 0.01, ***P < 0.001, and ****P < 0.0001.

Although ZIKV infection of AXL KO cells was significantly reduced, a low level of infection was still detectable, suggesting the possibility of selecting and enriching mutant viruses that infect these cells in an alternative AXL-independent manner. Indeed, continued passaging of AXL KO cells exposed to ZIKV ^PRV^ generated a mutant virus, designated as 1ZS3 (Fig. 4A), which achieved a much higher infection rate than the WT virus in the AXL KO cells while retaining similar infection efficiency in the WT SNB-19 cells. We produced the 1ZS3 virus from both SNB-19 and Vero cells and found that the AXL independence held in both cases (Fig. 4B through F).

To identify viral mutations responsible for the reduced AXL dependence of the 1ZS3 mutant virus, we performed two additional independent selections in the AXL KO cells and then sequenced the resistant virus from each selection. Although many mutations were present in each resistant virus, only one single mutation, H83R in the prM gene, was common in all of the resistant viruses selected from three independent experiments. Of note, a similar passage of the virus in the WT SNB-19 cells did not result in the appearance of this mutation (data not shown). Next, we investigated the effect of H83R in a defined genetic background of ZIKV ^FSS^ K265E (54) by comparing the infection efficiency of AXL KO cells with or without the H83R mutation. As shown in Fig. 4G through I, we found a more than 10-fold increase of both infection rate and virus titer of the KO cells by the H83R/K265E virus compared with the K265E virus, indicating that the H83R mutation in prM region is responsible for the observed AXL-independent phenotype. The H83R mutation also resulted in a slightly less than twofold but statistically significant increase in virus titer in the SNB-19 cells, consistent with a previous publication in which this mutation was among the mutations that can increase the yield of a ZIKV vaccine strain (54).

### The H83R mutation impacts virion structure but not prM cleavage or IFN signaling

**Fig 5:**
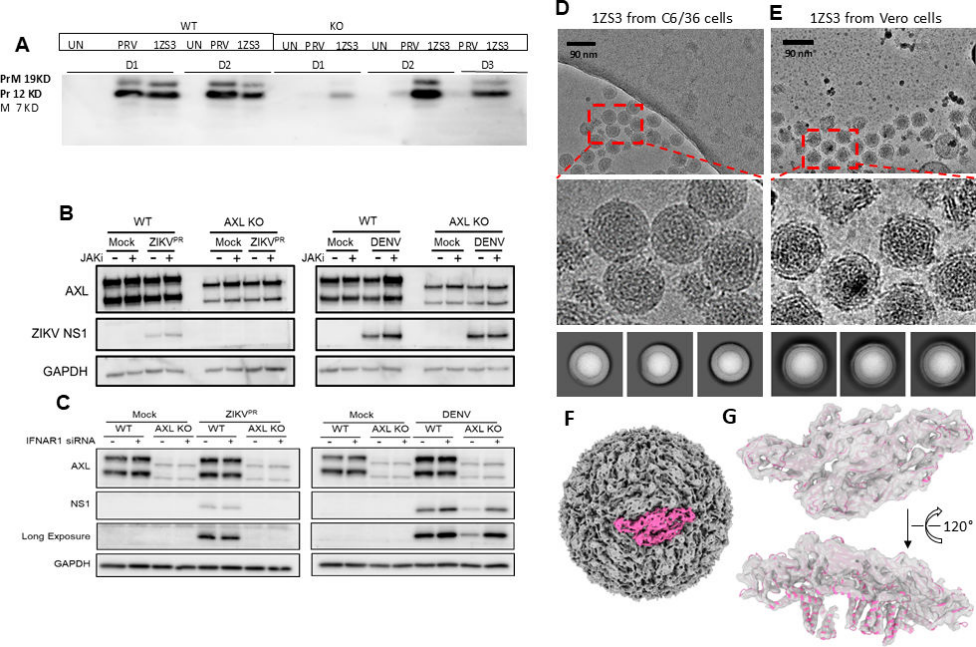
The 1ZS3 virus does not alter prM cleavage or IFN signaling but impacts virion maturation. (**A**) Western blot of cell lysate of human glioblastoma derived cell line (SNB) WT and AXL KO cells after 48 hpi (WT) and 72 hpi (AXL KO cells) of infection with WT or mutant virus. (**B**) ZIKV infection of AXL KO SNB-19 cells is not rescued by JAKi treatment. Representative western blots of WT or AXL KO SNB-19 cells that were treated with 1 mM JAKi at 1 h prior to ZIKV (MOI 0.5) or DENV (MOI 0.5) infection. (**C**) ZIKV infection of AXL KO SNB-19 cells is not rescued by siRNA knockdown of IFNAR1. Representative western blots of WT or AXL KO SNB-19 cells that were transfected with IFNAR siRNA for 48 h prior to ZIKV (MOI 0.5) or DENV (MOI 0.5) infection. (**D and E**) CryoEM characterization of ZIKV 1ZS3. CryoEM micrograph (top), zoom-in view (middle), and 2D class averages (bottom) of 1ZS3 virion purified from C6/36 cells (**D**) or Vero cells (**E**). (**F**) CryoEM density map of 1ZS3 purified from C6/36 cells. An asymmetric unit of the icosahedral structure was highlighted in pink. (**G**) Zoom-in views of the asymmetric unit densities in (**F**) fitted with the atomic model of the WT ZIKV (PDB 5IRE).

Given the location of H83 relative to the furin cleavage site, we performed western blots to examine the cleavage pattern of prM. No difference was observed between the WT and 1ZS3 viruses in this regard (Fig. 5A). We next investigated a distinct mechanism by which the H83R mutation may govern AXL dependency for ZIKV infection of SNB-19 cells. A recent report suggested that AXL permits ZIKV infection of astrocytes through the suppression of IFN signaling pathways (55); thus, we reasoned that if this mechanism is active in the SNB-19 cells, we can then determine whether H83R leads to reduced IFN activation and thus negating the need for AXL-mediated suppression. Treatment of WT SNB-19 cells with a JAK inhibitor (JAKi) prior to infection only minimally increased viral protein expression following ZIKV or DENV infection (Fig. 5B). Importantly, treatment with either JAKi or small interfering RNA (siRNA) targeting the interferon alpha receptor 1 (IFNAR1) did not rescue ZIKV infection in the AXL KO cells (Fig. 5C) in contrast to the results from the astrocytes (55). These data indicate that the AXL’s function in mediating ZIKV infection of these glioblastoma cells is largely independent of the IFN suppression mechanism and rather more likely to be associated with virion attachment, a step that can be influenced by virion maturation. Because of the important function of prM in virion assembly and maturation, we next carried out a structural study to compare the structures of WT and 1ZS3 virions. Interestingly, the 1ZS3 virions from Vero cells had a scrambled surface layer, and we were not able to get a converged cryoelectron microscopy (cryoEM) reconstruction with either ab initio model or using matured or immature structures of WT ZIKV as initial models (Fig. 5D and E). In contrast, 1ZS3 from C6/36 cells had essentially the same structure as the matured WT ZIKV (Fig. 5F and G) (56–58).

## DISCUSSION

Here, we report that AXL is an important host factor for ZIKV infection in glioblastoma cells as identified by a genome-wide CRISPR/Cas9 screen. A single mutation in the prM gene significantly reduced the dependence on AXL for infection. The mechanism by which ZIKV utilizes AXL for infection in the SNB-19 cells does not require innate immune suppression by AXL but likely relates to virion structural features that influence attachment during the subsequent rounds of infection. We found that the difference in AXL dependency between ZIKV and DENV tracks with the structural proteins, the infectivity difference between WT and AXL-independent viruses is more apparent during later time points of infection, and the H83R mutation in the prM gene responsible for reduced AXL-dependence alters virion structure.

Various molecular mechanisms have been proposed for the role of PS receptors in flavivirus infection. When AXL was first identified as an entry factor for DENV, mutations of both Gas6 and AXL which abolished PS-binding and Gas6-binding, respectively, prevented AXL-dependent enhancement of DENV infection (39). Further work with ZIKV found that sequestration of Gas6 by an AXL decoy receptor inhibited infection. Together, these early experiments suggest that the bridging function of Gas6 is essential for AXL-mediated viral entry (31). However, it is also possible that this binding is required not for virus internalization, but rather for AXL RTK (receptor tyrosine kinase) signaling (55). This is further complicated by work suggesting that the cell type used for DENV and WNV production greatly influences the usage of AXL, potentially due to differences in the exposure of PS on the virion membrane and the capacity of the virion to engage Gas6 (25). Of note, the accessibility of PS on the virion surface is still controversial (51, 53–55, 59, 60), and our results show that neither the 16,681 nor D2Y98P strains of DENV have the same AXL dependency as the three ZIKV strains tested (MR766, PRVABC59, and FSS), regardless of virus production source. Interestingly, the histidine residue at position 83 in ZIKV, which lies 11 amino acids upstream of the pr-M cleavage site for the furin protease, is only present in ZIKV and SPONV but not in other flaviviruses including DENV (types 1–4), WNV, JEV, or YFV. This is consistent with the notion that this histidine residue plays an important role in governing a more stringent requirement of AXL dependence, and its mutation into an arginine relaxes this requirement, likely to the level of other flaviviruses. Whether the downstream effects of the mutated residue that is not present in the mature virion can influence the level of PS exposure on the virions or AXL-binding remains to be determined.

There is still a low level of ZIKV infection which increases in a dose-dependent manner even in our AXL KO SNB-19 cells. We consider it unlikely that this was due to residual levels of AXL, as ZIKV infection still occurs in cell clones where both western blots and genomic sequencing clearly demonstrate a clean KO. It is possible that an alternative mechanism is at play for ZIKV infection in the absence of AXL, and this mechanism can be the same for AXL-independent infection by DENV. While other TAM and TIM receptors have been implicated in the enhancement of both ZIKV and DENV infections (39, 61), Tyro3, Mer, and TIM-1 are not expressed in SNB-19 cells. In addition, their ectopic expression did not increase WT ZIKV infection in the AXL KO cells. As a result, we consider that mutants gaining usage of these as unlikely as well. Other entry factors and putative receptors have been implicated in flavivirus infection, such as C-type lectin receptors (62), integrins (10, 63–65), and surface sialic acid (66), so it is conceivable that the mutation enables the engagement of some of these cell surface molecules.

Efficient furin cleavage of prM is required for maturation of flaviviruses including ZIKV and DENV (67–69), so a potential explanation for the altered structure of the H83R virion could be that the mutation impacts the rate of the cleavage, leading to a change in the virion maturation process. However, we did not detect any difference in the ratio of M protein to prM precursor protein between ZIKV WT or 1ZS3, either in the infected cells or in purified virions (Fig. 5A and data not shown). Alternatively, the change from a histidine to an arginine can influence the pH-dependent shedding of pr, which has been shown to be important for the proper interaction between M and E as a critical subsequent step of flavivirus maturation (70, 71). Histidine residues on E have been shown to be critical for membrane fusion as well (72), although that would be a step further down the pathway and well after the release of the pr fragment from the virion. Regardless of the specific mechanism, the impact of H83R on virion is not apparent in C6/36 cells which could be due to, among other possibilities, the differences in activities between human and mosquito furin-like proteases (73) or in the pH-dependence release of pr from the virions between Vero and C6/36 cells. The H83R mutation is one of the three mutations previously engineered to increase the yield of a vaccine strain (54), but it remains to be determined whether the overall increase in the infectivity of the virus is related to AXL usage or not.

In summary, our data reveal a link between prM function during virion assembly and PS receptor usage during entry attachment of progeny ZIKV virions and identify an important molecular determinant on prM that regulates virion maturation at a step prior to pr shedding. Additional detailed biochemical and structural characterizations of these new connections will significantly deepen our knowledge of flavivirus replications, particularly assembly, maturation, and entry.

## MATERIALS AND METHODS

### Cell lines

The human glioblastoma SNB-19 cell line was cultured in Roswell Park Memorial Institute (RPMI-1640) medium supplemented with 10% fetal bovine serum (FBS; Invitrogen) and incubated at 37°C in 5% CO_2_. *Aedes albopictus* C6/36 cells (ATCC) were cultured in Eagle’s minimum essential medium supplemented with 10% FBS and incubated at 28°C in 5% CO_2_. Vero cells (ATCC) were maintained in Dulbecco’s modified Eagle’s medium (DMEM) supplemented with 10% FBS and 1% non-essential amino acids at 37°C and 5% CO_2_.

### Flavivirus generation

ZIKV and DENV stocks were amplified in C6/36 cells at 28°C. Briefly, C6/36 cells cultured in T-75 flasks were incubated with ZIKV and DENV viral inoculum for 1 h at room temperature, then fresh media was added, cells were cultured for 7 days, and collected supernatant was filtered with a 0.45 µm filter and aliquoted and stored at −80°C. The plasmid constructs for ZIKV FSS13025, DENV-2 strain D2Y98P, CHV-I, CHV-II, NS1 K265E, and NS1 K265E Plus PrM H83R were obtained from Dr. Pei-yong Shi at The University of Texas Medical Branch at Galveston, Galveston, TX. ZIKV FSS13025, DENV-2 strain D2Y98P, CHV-I, and CHV-II virus stocks were prepared in C6/36 cells as previously described (53). K265E and K265E Plus H83R were amplified in Vero cells at 37°C. Vero cells cultured in T-75 flasks were incubated with the desired virus for 2 h at 37°C, then fresh media was added, cells were cultured until the cytopathic effect was seen in 80% of cells, and collected supernatant was filtered with a 0.45 µm filter and aliquoted and stored at −80°C.

### Infection and titration

For infection, cells were seeded in culture vessels 1 day before the experiment. The desired amount of virus stock was added to the cells at the indicated MOI. The cells were incubated for 2 h at 37°C with gentle shaking every 15 min before the inoculum was removed, and fresh medium was added. The titers of amplified viruses were determined by focus-forming unit (FFU). For the FFU assay, cells were blocked in PBTG (phosphate-buffered saline [PBS], 0.5% Tween-20, 1% bovine serum albumin [BSA], 0.2% BSA, and 5% normal goat serum) and then incubated overnight at 4°C with anti-flavivirus-envelope (Clone D1-4G2-4-15, produced from hybridoma ATCC HB-112). The following day, the cells were washed three times with PBS prior to incubation with goat-anti-mouse IgG horse radish peroxidase (HRP; Santa Cruz Biotechnology, Cat # sc-2005) for 1 h at room temperature and then washed three times with PBS prior to incubation with 3,3’-diaminobenzidine (DAB) peroxidase substrate (Vector Labs, Cat # SK-4100) for 10 min.

### Genome-wide CRISPR/Cas9 screening

The sgRNA library (GeCKO v2 Human Library; Addgene, Cat # 1000000048) was amplified from initial pooled stocks (library A and library B respectively) as previously described (39, 40). The library was then transfected into 293T cells using lipofectamine 2000 (Invitrogen, Cat # 11668019) and transfection plus reagent (Invitrogen, Cat # 11514015) to produce lentivirus. Lentivirus-containing supernatant was harvested at 48 h, 72 h, and 96 h post-transfection and pooled and filtered prior to aliquoting and storage at −80°C. Following collection, the library-containing lentivirus was functionally titered on SNB-19 cells through puromycin selection and quantification of cell survival. For screening purposes, SNB-19 cells were seeded, and approximately 2 × 10^8^ cells were transduced the following day with the GeCKO library lentivirus at an MOI of 0.3. Puromycin was introduced to the cells at 48 h post-transduction and maintained for 2 weeks. An untransduced flask was simultaneously treated with puromycin as a timing control. Following puromycin selection, transduced SNB-19 cells were infected with ZIKV (strain MR766) at an MOI of 1 for 3 weeks. Again, an untransduced population of cells was infected with ZIKV concurrent to the selected population as a control for virus-induced cell death. At the end of the 3 weeks, surviving cell colonies were harvested for subsequent genomic DNA extraction and analysis. An additional batch of transduced cells was collected following puromycin selection to determine the library transduction efficiency. These actions were collectively performed in duplicate as two biological replicates.

### Genomic DNA extraction, sequencing, and data analysis

Collected cell pellets were first resuspended in 100 µL of PBS prior to mixing with DNA extraction solution (100 mM NaCl, 10 mM TrisCl pH 8, 25 mM EDTA pH 8, 0.5% SDS, and 0.1 mg/mL proteinase K added fresh). Resuspended cell mixtures were heated at 50°C for 2–4 h prior to extraction with phenol/chloroform/isoamyl alcohol (Invitrogen, Cat# 15593031). After a final extraction with chloroform, 1/10 vol of 3 M NaOAc was added to the recovered aqueous phase followed by 2 volumes of 100% ethanol. The DNA was precipitated at −20°C overnight, recovered by centrifugation, washed with 70% ethanol, and finally resuspended in Tris-EDTA (TE) buffer.

Samples were prepared for Next Generation Sequencing via PCR amplification utilizing primers designed to target regions flanking the inserted sgRNA site as previously described (39). Following a second PCR to attach HiSeq flowcell adapters to the amplified constructs, sequencing reactions were run on an Illumina HiSeq 2500, and the downstream output was analyzed for significant candidate gene targets using the Model-based Analysis of Genome-wide CRISPR/Cas9 Knockout (MAGeCK) computational tool (74).

### AXL CRISPR knockout generation and validation

To validate the GeCKO library screening results, one of the sgRNA constructs targeting AXL was introduced back into SNB-19 cells through lentivirus packaging of the pLentiCRISPRv2 backbone (Genscript, gRNA target sequence CTGCGAAGCCCATAACGCCA) and transduction. Two weeks after transduction, cells were enriched for AXL-negative populations through FACS (BD FACSAria III) and single-cell cloning. Single cell-derived populations were screened for AXL expression via western blotting with goat anti-AXL (R&D Systems, Cat# AF154). For selected clones, genomic DNA was extracted as described in the previous section. PCR primers were designed to flank each respective sgRNA target site and amplify the cut region (GTGAGACAGTGTGTGTGC and GAACCAAGTTTGGGAGTCTC) for TOPO cloning (Invitrogen, Cat# 451641) and sequenced alongside untransduced SNB-19 cells (Applied Biosystems 3730 Genetic Analyzer) for cut-site analysis.

Lentiviral constructs expressing AXL and the other PS receptors were inserted in the pUltra backbone expressing eGFP and an HA tag at the C terminus of the cDNA. Lentiviral transduction of the cDNA clones was done similarly as the CRISPR/Cas9 lentiviruses. The transduced cells were sorted by FACS and showed more than 95% of the population being GFP positive. The synonymous mutation, which renders the AXL cDNA resistant to the original sgRNA in the KO cells, in the M2 version of the AXL cDNA constructs was engineered using the Quick-change lightning site-directed mutagenesis kit (Agilent Cat#210519).

### Generation of 1ZS3 virus

SNB-19 AXL KO cells were infected with ZIKV ^PRV^ at MOI 5 and then split when the cells were confluent to continue the passaging for 30–32 days. We used supernatant from the cells at the end of the passaging to infect naïve AXL KO cells for 2 h, followed by changing into fresh media and supernatant collection at 48–72 hpi. This collection and reinfection of naïve KO cells were performed for three rounds, after which the supernatant was collected and named 1ZS3. For parallel passaging in the WT background, we infected the SNB-19 cells with ZIKV ^PRV^ at MOI 0.5 and collected supernatant after 48–72 hpi. Similar collection and reinfection of naïve WT cells were performed for three rounds, and the final virus collected is named PRVS3.

### Viral RNA extraction, cDNA preparation, sequencing, and data analysis

Viral RNA of 1ZS3 and PRVS3 was extracted by using the Viral RNA Qiagen kit (Cat # 52904) from the supernatant of infected AXL KO and WT cells, respectively. cDNA was prepared by SuperScript III First-strand synthesis system for RT-PCR (Thermo Fisher Scientific, Cat# 18080–051). We used 21 primer sets to amplify the whole genome, and each PCR fragment was TOPO cloned (Invitrogen, Cat # 451641) and sequenced by Sanger sequencing method.

### Western blotting

SNB-19 cells were directly lysed in Laemmli sample buffer and immediately boiled for 10 min. Proteins in the samples were separated by 10% or 12% SDS-PAGE and transferred to polyvinylidene fluoride (PVDF) membrane (Millipore, Cat # IPVH00010). The membranes were blocked in 10% milk in phosphate-buffered saline with Tween (PBST) (1× PBS solution containing 0.5% Tween-20 and 1% BSA) and then incubated either at room temperature for 1 h or overnight at 4°C with the following primary antibodies: anti-ZIKV NS1 (BioFront Technologies, Cat # BF-1225–36), anti-DENV NS1 (BioFront Technologies, Cat # BF-1191–46), rabbit-anti-AXL (Cell Signaling Technology, Cat # C89E7), goat-anti-AXL (R&D Systems, Cat# AF154), anti-GAPDH (GeneTex Cat # GTX100118), or anti HA tag (Millipore sigma, Cat # H965B). Membranes were washed three times in PBST and then probed with HRP-conjugated secondary antibodies for 1 h at room temperature. After three PBST washes, a chemiluminescent HRP substrate (Millipore, Cat # WBKLS0500) was applied to detect protein on a Bio-Rad ChemiDoc MP Imaging System.

### Immunofluorescence

Cells were seeded on coverslips and fixed with 4% paraformaldehyde, permeabilized with phosphate-buffered saline with Triton-X-100 (PBT) (0.2% Triton-X-100 in PBS), and then blocked in PBTG (PBT, 0.2% BSA, and 5% normal goat serum). After blocking, cells were stained with anti-flavivirus envelope (Clone D1-4G2-4-15 produced from hybridoma ATCC HB-112). Slides were washed three times in PBS and then stained with a goat-anti-mouse IgG fluorescein isothiocyanate (FITC) secondary antibody (Sigma-Aldrich, Cat # F0257). Slides were washed three times in PBS and mounted using Vectashield mounting medium with 4’,6-diamino-2-phenylindole-fluorescent nucleic acid dye (DAPI) (Vector Laboratories H-1200) and then imaged using a Zeiss Axiovert 200M microscope. Collected images were analyzed and quantified using ImageJ (https://imagej.nih.gov/ij/).

### Flow cytometry analysis

WT or AXL KO SNB-19 cells were infected for 24 h. Cell pellets (1–3 million cells) were collected by trypsinization 24 h post-infection. Samples were washed with PBS and fixed with 4% paraformaldehyde for 10 min. For analysis by 2D flow cytometry, the samples were incubated either alone or in combination with the following antibodies: 0.15 µg goat-anti-AXL (R&D Systems, Cat # AF154), goat-anti TIM-1 (R&D Systems, Cat # AF1750), goat-anti-Tyro3 (R&D Systems, Cat # AF859), goat-anti- MerTK (R&D Systems, Cat # AF891), normal goat IgG (R&D Systems, Cat # AB-108-C), or mouse-anti-flavivirus envelope (Clone D1-4G2-4-15 produced from hybridoma ATCC HB-112) in a PBST for 1 h. Samples were washed with PBT solution and incubated with 1 µg goat-anti-mouse IgG FITC (Sigma-Aldrich, Cat # F0257) or donkey-anti-mouse IgG Alexa Fluor 488 (Abcam, Cat # ab150105) in PBT for 30 min. After washing with PBT, samples were analyzed using a BD FACSCanto machine (BD Biosciences). Cell cycle plots were generated using FlowJo software (FlowJo, LLC).

### Small interfering RNA and JAK inhibitor experiments

SNB-19 cells were seeded such that cells were 30% confluent the next day and transfected 24 h post-seeding with SMARTpool: ON-TARGETplus AXL siRNA (Dharmacon, Cat # L-003104–00) or IFNAR1 siRNA (Dharmacon, Cat # L-020209–00) using the Lipofectamine RNAiMAX reagent (Invitrogen, Cat # 13778030). At 48 h post-transfection, cells were infected with ZIKV or DENV (MOI 0.5). For JAKi experiments, JAKi (Calbiochem, Cat # 420097) was added 1 h prior to infection at a final concentration of 1 mM. Samples were collected at 24 h post-infection for western blot analysis.

### Virus purification for structural analysis

ZIKV ^PRV^ and 1ZS3 mutant stocks were prepared by growing the virus on Vero monolayers. For virus purification, ~1 × 10^9^ of Vero cells grown in DMEM medium supplemented with 2% FBS were infected with the virus at MOI of 0.1. After 60–72 h, culture supernatant was collected, filtered, and precipitated with 8% PEG8000 overnight at 4°C, and the precipitate containing virions was collected after centrifugation at 10,000 × *g* for 50 min on a 20% sucrose cushion. The precipitate was resuspended in TE buffer (10 mM Tris-HCl pH 8, 150 mM NaCl, and 1 mM EDTA) and subjected to density-gradient centrifugation using a 10%–50% sucrose gradient at 100,000 × *g* for 2 h at 4°C. The virus fraction was collected from the gradient, concentrated, and buffer exchanged into TE buffer using Amicon centrifugal filters (Sigma, # UFC500396). The purity of the final virus preparation was analyzed using SDS gel electrophoresis.

### Cryo-electron microscopy

To prepare cryoEM sample, a 3.5 µL aliquot of the purified virus was applied to a 300-mesh Quantifoil R1.2/1.3 Cu grid pretreated with glow-discharge, blotted with filter paper in a Vitrobot Mark IV (Thermo Fisher Scientific), and plunge-frozen in liquid ethane. The grid was then transferred to a Titan Krios microscope equipped with Gatan BioQuantum K3 imaging filter and camera (Thermo Fisher Scientific) for data collection. Movie stacks were recorded at 81,000× magnification, corresponding to a pixel size of 0.53 Å/pixel at the super-resolution mode of the camera. A defocus range of −1.0 µm to −1.8 µm was set. A total dose of 50 e^−^/Å2 of each exposure was fractionated into 50 frames. Data processing was done with cryoSPARC (75) following the standard single-particle analysis scheme.
